# 11.4 AEROBIC EXERCISE ENHANCES COGNITIVE TRAINING EFFECTS IN FIRST EPISODE SCHIZOPHRENIA: COGNITIVE AND FUNCTIONAL GAINS AND PROMISING BIOLOGICAL MECHANISMS OF ACTION

**DOI:** 10.1093/schbul/sby014.042

**Published:** 2018-04-01

**Authors:** Keith Nuechterlein, Sarah McEwen, Joseph Ventura, Kenneth Subotnik, Luana Turner, Michael Boucher, Laurie Casaus, Jacqueline Hayata

**Affiliations:** University of California, Los Angeles

## Abstract

**Background:**

The search for treatments to remediate cognitive deficits and their functional outcome consequences remains a critical frontier in schizophrenia. Cognitive training and aerobic exercise both show promising moderate impact on cognition and everyday functioning. Aerobic exercise is hypothesized to increase brain-derived neurotrophic factor (BDNF) and thereby stimulate neurogenesis and synaptic plasticity, leading to increased learning capacity. Systematic cognitive training should take advantage of increased learning capacity and be more effective when combined with aerobic exercise.

**Methods:**

In a recently completed randomized controlled trial, we examined the impact of a 6-month program of Cognitive Training & Exercise (CT&E) compared to Cognitive Training alone (CT) in 47 first-episode schizophrenia outpatients. All participants were provided the same Posit Science computerized cognitive training, four hours/week, using BrainHQ and SocialVille programs. The CT&E group also participated in total body circuit training exercises to enhance aerobic conditioning. The exercise intensity was in the 60–80% of aerobic capacity range, combining clinic and home-based exercise for a target of 150 minutes per week.

**Results:**

Mixed model analyses demonstrate that the MATRICS Consensus Cognitive Battery Overall Composite improves significantly more by 3 months with CT&E than with CT alone (6.6 vs. 2.2 T-score points, p<.02). Work/school functioning improves substantially more with CT&E than with CT alone by 6 months (p<.001). BDNF is a promising mechanism of action, improving even after 2 weeks and predicting the amount of cognitive gain at 3 months. The magnitude of cognitive gain by 3 months predicts the amount of work/school functioning improvement at 6 months, suggesting a cascade of effects. Analyses by Dr. McEwen show differential increases in cortical thickness in the left dorsal lateral prefrontal gyrus (p=.02) and right superior frontal gyrus (p=.02) over 6 months and increased functional connectivity in the central executive network (p=.04) with CT&E compared to CT alone and correlations of these increases with cognitive and functional outcome gains.

**Discussion:**

We conclude that aerobic exercise significantly enhances the impact of cognitive training on cognition, functional outcome, and frontal cortical thickness in first-episode schizophrenia and that BDNF is a promising mechanism of action for these effects.

